# Supplementary material for: Delirium and its association with short-term outcomes in younger and older patients with acute heart failure
Source: PLoS One. 2022 Jul 26;17(7):e0270889. doi: 10.1371/journal.pone.0270889 (PMC9321444; doi:10.1371/journal.pone.0270889)
Supplement: S1 Table — IQR, interquartile range; SBP, systolic blood pressure; BUN, blood urea nitrogen; ED, emergency department; ICU, intensive care unit. (DOCX) [file pone.0270889.s001.docx]

**S1 Table.** Patient characteristics of patients who were in the analysis cohort, had missing cognition and other covariate data, and were lost to follow-up**.** IQR, interquartile range; SBP, systolic blood pressure; BUN, blood urea nitrogen; ED, emergency department; ICU, intensive care unit.

| **Variable** | **Patients included in the analysis**  **n=1,044** | **Patients with missing cognition data**  **n=47** | **Patients with missing covariate data**  **N=38** | **Patients with missing follow-up data**  **n=79** |
| --- | --- | --- | --- | --- |
| Median (IQR) Age, years | 61 (52, 71) | 66 (55, 80) | 69 (59, 80) | 60 (49, 79) |
| Female, n (%) | 462 (44.3%) | 22 (46.8%) | 16 (42.1%) | 38 (48.1%) |
| White  Non-White Race, n (%)  American Indian  Asian  Black  Pacific Islander  Other  Unknown | 366 (35.1%)  7 (0.7%)  4 (0.4%)  652 (62.5%)  3 (0.3%)  10 (1.0%)  2 (0.2%) | 33 (70.2%)  0 (0.0%)  0 (0.0%)  14 (28.8%)  0 (0.0%)  0 (0.0%)  0 (0.0%) | 17 (44.7%)  0 (0.0%)  1 (2.6%)  20 (52.6%)  0 (0.0%)  0 (0.0%)  0 (0.0%) | 5 (6.3%)  1 (1.3%)  2 (2.5%)  71 (89.8%)  0 (0.0%)  0 (0.0%)  0 (0.0%) |
| Median (IQR) Education, years | 12 (12, 13) | 12 (11, 13) | 13 (13, 15) | 12 (12, 13) |
| Median (IQR) HF Mortality Risk | 0.02 (0.01, 0.03) | 0.02 (0.01, 0.04) | 0.03 (0.02, 0.04) | 0.01 (0.01, 0.03) |
| Median (IQR) SBP, mmHg | 148 (127, 171) | 150 (125, 173) | 143 (128, 158) | 148 (128, 174) |
| Median (IQR) Heart Rate, beats  per minute | 90 (77, 103) | 88 (75, 99) | 88 (77, 101) | 92 (79, 107) |
| Median (IQR) BUN, mg/dL | 22 (16, 32) | 27 (18, 34) | 26 (19, 40) | 20 (14, 32) |
| Ejection Fraction < 40%, n (%) | 380 (36.4%) | 7 (14.9%) | 13 (34.2%) | 29 (36.7%) |
| Past history, n (%) |  |  |  |  |
| Myocardial infarction | 300 (28.7%) | 16 (34.0%) | 10 (26.3%) | 25 (31.7%) |
| Hypertension | 917 (87.8%) | 40 (85.1%) | 31(81.6%) | 71 (89.9%) |
| Diabetes Mellitus | 477 (45.7%) | 21 (44.7%) | 13 (34.2%) | 35 (44.3%) |
| Dyslipidemia | 537 (51.4%) | 20 (42.6%) | 21 (44.7%) | 30 (38.0%) |
| Chronic Kidney Disease | 359 (34.4%) | 15 (31.9%) | 17 (44.7%) | 20 (25.3%) |
| Dialysis Dependent | 55 (5.3%) | 5 (10.6%) | 3 (7.9%) | 5 (6.3%) |
| Pulmonary Hypertension | 84 (8.1%) | 0 (0.0%) | 1 (2.6%) | 5 (11.39%) |
| Discharged Home from ED | 76 (7.3%) | 3 (6.4%) | 1 (2.6%) | 10 (12.7%) |
| Ever Admitted to an ICU | 103 (9.8%) | 11 (23.4%) | 6 (15.8%) | 1 (1.3%) |
