# Supplementary material for: Delirium and its association with short-term outcomes in younger and older patients with acute heart failure
Source: PLoS One. 2022 Jul 26;17(7):e0270889. doi: 10.1371/journal.pone.0270889 (PMC9321444; doi:10.1371/journal.pone.0270889)
Supplement: S2 Table — Heart failure (HF) mortality risk was estimated using age, heart rate, systolic blood pressure, and blood urea nitrogen. Delirium was determined using the brief Confusion Assessment Method (bCAM). IQR, interquartile range; SBP, systolic blood pressure; BUN, blood urea nitrogen; ED, emergency department; ICU, intensive care unit. (DOCX) [file pone.0270889.s002.docx]

**S2 Table.** Patient characteristics stratified by 30-day composite outcome status.. Heart failure (HF) mortality risk was estimated using age, heart rate, systolic blood pressure, and blood urea nitrogen. Delirium was determined using the brief Confusion Assessment Method (bCAM). IQR, interquartile range; SBP, systolic blood pressure; BUN, blood urea nitrogen; ED, emergency department; ICU, intensive care unit.

| **Variable** | **30-day Composite Endpoint Present**  **N=446** | **30-day Composite Endpoint Absent**  **N=598** | **P**-**value** |
| --- | --- | --- | --- |
| Median (IQR) Age, years | 63 (54, 73) | 60 (50, 79) | 0.0017 |
| Female, n (%) | 199 (44.6%) | 263 (44.0%) | 0.8371 |
| White, n (%)  Non-White Race, n (%)  American Indian  Asian  Black  Pacific Islander  Other  Unknown | 179 (40.1%)  267 (59.9%)  1 (0.2%  1 (0.2%)  258 (57.9%)  1 (0.2%)  4 (1.1%)  1 (0.2%) | 187 (31.3%)  411 (57.7%)  6 (1%)  3 (0.5%)  394 (65.9%)  2 (0.3%)  5 (0.8%)  1 (0.2%) | 0.0587 |
| Median (IQR) Education, years | 12 (10, 12) | 12 (10, 12) | 0.7553 |
| Median (IQR) Short Blessed Test | 6 (2, 12) | 4 (2, 10) | 0.0033 |
| Median (IQR) HF Mortality Risk | 0.02 (0.01, 0.04) | 0.01 (0.01, 0.02) | <0.0001 |
| Median (IQR) SBP, mmHg | 142 (119, 166) | 150 (131, 175) | <0.0001 |
| Median (IQR) Heart Rate, beats per min | 90 (77, 103) | 91 (77, 103) | 0.6885 |
| Median (IQR) BUN, mg/dL | 24 (18, 37) | 20 (15, 28) | <0.0001 |
| Ejection Fraction < 40%, n (%) | 154 (34.5%) | 226 (37.8%) | 0.2783 |
| Past history, n (%) |  |  |  |
| Myocardial infarction | 127 (28.5%) | 173 (28.9%) | 0.8725 |
| Hypertension | 385 (86.3%) | 598 (57.3%) | 0.1967 |
| Diabetes Mellitus | 208 (46.6%) | 269 (45.0%) | 0.5957 |
| Dyslipidemia | 241 (54.0%) | 296 (49.5%) | 0.1468 |
| Chronic Kidney Disease | 163 (35.5%) | 196 (32.8%) | 0.2045 |
| Dialysis dependent | 21 (7%) | 24 (4%) | 0.0356 |
| Pulmonary hypertension | 41 (9.2%) | 43 (7.2%) | 0.2394 |
| Discharged Home from ED | 15 (3.4%) | 61 (10.2%) | <0.0001 |
| Ever admitted to an ICU | 79 (17.7%) | 62 (10.4%) | 0.0006 |
