# Supplementary material for: Delirium and its association with short-term outcomes in younger and older patients with acute heart failure
Source: PLoS One. 2022 Jul 26;17(7):e0270889. doi: 10.1371/journal.pone.0270889 (PMC9321444; doi:10.1371/journal.pone.0270889)
Supplement: S3 Table — Initially, older age*delirium and older age*cognitive impairment without delirium interaction terms were incorporated in the multivariable logistic regression model. Because, their p-values were 0.47 and 0.31, respectively, these interaction terms were removed from the model to maintain parsimony. (DOCX) [file pone.0270889.s003.docx]

**S3 Table.** Multivariable logistic regression model for the primary composite outcome of 30-day all-cause death, 30-day all-cause rehospitalization, or hospitalization > 7 days. Initially, older age*delirium and older age*cognitive impairment without delirium interaction terms were incorporated in the multivariable logistic regression model. Because, their p-values were 0.47 and 0.31, respectively, these interaction terms were removed from the model to maintain parsimony.

| **Covariate** | **Odds Ratio (95%CI)** |
| --- | --- |
| Delirium | 1.64 (1.02 - 2.64) |
| Cognitive impairment without delirium | 0.91 (0.66 - 1.24) |
| Old | 1.03 (0.76 - 1.38) |
| Acute heart failure mortality risk score | 1.59 (1.35 - 1.88) |
| Education, years | 0.98 (0.92 - 1.04) |
| Ejection fraction < 40% | 0.75 (0.56 - 1.01) |
| Unknown ejection fraction | 0.78 (0.54 - 1.13) |
| Male sex | 1.02 (0.78 - 1.33) |
| Black race | 0.92 (0.69 - 1.23) |
| Myocardial infarction | 0.84 (0.66 - 1.06) |
| Hypertension | 0.80 (0.53 - 1.21) |
| Diabetes | 1.01 (0.78 - 1.33) |
| Chronic Kidney Disease | 0.91 (0.68 - 1.22) |
| Dialysis | 1.62 (0.89 - 2.93) |
| Dyslipidemia | 1.15 (0.87 - 1.51) |
| Pulmonary hypertension | 1.38 (0.86 - 2.21) |
