# Supplementary material for: Delirium and its association with short-term outcomes in younger and older patients with acute heart failure
Source: PLoS One. 2022 Jul 26;17(7):e0270889. doi: 10.1371/journal.pone.0270889 (PMC9321444; doi:10.1371/journal.pone.0270889)
Supplement: Attachment — Submitted filename: Delirium HF - Response Letter 2022-06-02.docx [file pone.0270889.s006.docx]

Enrico Mossello, MD, PhD

Academic Editor

*PLOS ONE*

June 1, 2022

RE: PLOS ONE resubmission titled " Delirium and Its Association with Short-term Outcomes in Younger and Older Patients with Acute Heart Failure"

Dear Editors:

Thank you for the detailed reviews and for allowing us to revise and resubmit this manuscript. We have addressed all the suggestions/criticisms below and we hope that you find our revisions satisfactory. The details of our changes are provided in the subsequent pages; our responses are preceded by “***”. Thank you again for allowing us the opportunity to revise and resubmit this manuscript. If you have any questions, please don’t hesitate to contact me by phone (615-260-0086) or by e-mail.

Sincerely,

Jin Ho Han, MD, MSc

Associate Professor of Emergency Medicine

Critical Illness, Brain Dysfunction, and Survivorship (CIBS) Center

Vanderbilt University Medical Center

Geriatric Research, Education, and Clinical Center (GRECC)

Tennessee Valley Health Care System

jin.h.han@vumc.org

Please describe better patient selection ("convenience sample" is not sufficient) and give an estimate of exclusion rate.

***Thank you for this suggestion. We added an Enrollment Flow Diagram (Figure 1) to better characterize our exclusions. We also added the following (see underlined) to the Results on Page 7:

“Of the 20,883 patients who presented with AHF in the participating EDs, 1500 patients participated in the EMROC Registry (**Figure 1**), and 1208 patients had an adjudicated diagnosis of AHF. Of these, 47 patients were excluded because they had had missing SBT or bCAM assessments, 38 patients were excluded because of missing covariate data, and 79 were excluded because they were lost to follow-up.”

Describe better among-group differences of HF mortality risk (the highly significant difference are not apparent from data in table 1).

***Per your suggestion, we have added **Supplemental Table 2** which stratifies patient characteristics by 30-day composite outcome status. In the Results section (Page 10), we also added the following text:

“Patient characteristics stratified by 30-day composite outcome status can be seen in **Supplementary Table 2**. Patients who had the 30-day composite outcome were older, had higher SBT scores and mortality risk, and were more likely to be on dialysis prior to enrollment, be admitted to the hospital, and be admitted to the ICU.”

Include a supplementary analysis regarding the difference between older and younger patients and briefly discuss the reason of the lack of prognostic meaning of age and the similar prevalence of delirium between groups, both unexpected

***Thank you for this suggestion. We have added **Supplemental Table 4**. Patient characteristics stratified by older (> 65 years) versus younger (<65 years) at enrollment. We also added the following paragraph to the discussion section to provided potential reasons:

“Surprisingly, younger age was not protective as the proportion of AHF patients with delirium were similar between older and younger patients. Age also did not modify the association between delirium and the 30-day composite outcome. Younger patients were more likely to have an ejection fraction < 40% (Supplementary Table 4) which can increase the patient’s vulnerability to developing delirium due to brain hypoperfusion [26, 27]. Reduced ejection fraction also is a powerful predictor of all-cause mortality and may potentiate delirium’s effect on long-term outcomes [28]. Younger patients with AHF were also more likely to self-identify as Black who are at higher risk for mortality and morbidity, and higher risk for heart failure-related hospitalizations compared with their non-Black counterparts [29, 30]. This is likely driven by health disparities and negative social determinants of health. Black patients with jeart failure are more likely to have poorer access to health care and have a decreased likelihood of receiving guideline-recommended heart failure therapies [31, 32]. Black race is also associated with poorer heart failure disease self-management and decreased medication adherence [33], which can lead to AHF and subsequent hospitalizations. They are also more likely to have comorbidities such as diabetes, hypertension, and obesity which may further complicate disease self-management and medication adherence [34]. The complex interplay among age, health disparities, and social determinants of health may explain our findings and would have important implications for potential interventions aimed at improving HF outcomes in diverse patient populations.”

Among limitations, you should include the lack of information regarding functional status (frailty and/or disability).

***Thank you for pointing this out. We added the following sentence to the limitations section:

“We also did not account for pre-illness comorbidity burden, frailty or disability which may have led to additional residual confounding.”

Please consider to compute a comorbidity index to adjust for in a sensitivity analysis.

***Our registry collected a past history of myocardial infarction, hypertension, diabetes mellitus, chronic kidney disease, dialysis, dyslipidemia, pulmonary hypertension, and ejection fraction < 40%. These were adjusted in the multivariable models since we had sufficient degrees of freedom. Unfortunately, we did not collect all the variables necessary to calculate the Charlson or Elixhauser comorbidity burden and this is a limitation. We added the following sentence to the limitations section:

“While we adjusted for a past history of myocardial infarction, hypertension, diabetes mellitus, chronic kidney disease, dialysis, dyslipidemia, pulmonary hypertension, we did not capture all the comorbidities necessary to calculate the Charlson or Elixhauser comorbidity burden. We also did not account for pre-illness frailty or disability. The lack of adjustment for these covariates may have led to additional residual confounding.

Moreover "cognitive impairment" does not represent premorbid cognitive impairment, also in subjects without delirium, and likely represents a mix between premorbid cognitive impairment, subsyndromal delirium and "false positives" (low education, anxiety, etc). Please discuss.

***In the limitations, the following sentence were added:

“Even in patients without delirium, the SBT may not have accurately reflected pre-illness cognition. Some of these patients may have had subsyndromal delirium or their scores were falsely low due to anxiety or multiple distractions (i.e.,monitor alarms) commonly seen in the ED environment.”

Editorial Office Comments

1. Thank you for stating the following financial disclosure:

"This project was partially supported by the NCATS/NIH under award number UL1 TR000445. Drs. Han and Collins receive funding from the Geriatric, Research, Education, and Clinical Center (GRECC)."

Please state what role the funders took in the study. If the funders had no role, please state: "The funders had no role in study design, data collection and analysis, decision to publish, or preparation of the manuscript."

If this statement is not correct you must amend it as needed.

Please include this amended Role of Funder statement in your cover letter; we will change the online submission form on your behalf.

*** The funders had no role in study design, data collection and analysis, decision to publish, or preparation of the manuscript. We have added this to the cover letter. Thank you for changing the online submission form on our Behalf.

2. One of the noted authors is a group or consortium [Emergency Medicine Research and Outcomes Consortium Investigators]. In addition to naming the author group, please list the individual authors and affiliations within this group in the acknowledgments section of your manuscript. Please also indicate clearly a lead author for this group along with a contact email address.

***We have added the following to the Acknowledgements:

“The Emergency Medicine Research and Outcomes Consortium (EMROC) Investigators consist of Drs. Sean Collins (Vanderbilt University Medical Center); Peter Pang (Indiana University and Eskenazi Hospitals), Phillip Levy (Detroit Receiving and Sinai Grace Hospitals); and Gregory Fermann (University of Cincinnati Medical Center). Dr. Sean Collins (e-mail: sean.collins@vumc.org) leads the EMROC Investigators.”
